# Acute intraventricular hemorrhage triggered by coughing following return from high altitude: a case report and literature review

**DOI:** 10.3389/fnins.2026.1743120

**Published:** 2026-02-17

**Authors:** Pengpeng Li, Yangyang Gao, Junfeng Li, Shaohua Lin, Zehong Zhang, Lei Luo, Wei Liu

**Affiliations:** Xi'an Aerospace Hospital of Northwest University, Yanta District, Xi'an, Shanxi, China

**Keywords:** case report, cough, de-acclimatization, high altitude, intraventricular hemorrhage

## Abstract

**Background:**

Exposure to high-altitude conditions can lead to significant physiological stress and elevate the risk of cerebrovascular incidents. Nevertheless, intraventricular hemorrhage (IVH) caused by a minor trigger such as coughing shortly after returning from brief high-altitude travel remains highly uncommon.

**Case Presentation:**

A 30-year-old woman with no significant prior medical history developed a sudden thunderclap headache and began vomiting right after a strong cough. This occurred on the day she came back to Xi'an (400 m) from a four-day visit to Xining (2,260 m), where she had experienced considerable sleep disruption. A non-contrast head CT scan showed bleeding inside the ventricular system. Subsequent CT angiography (CTA) did not detect any aneurysms or vascular malformations. She was successfully treated with a neuronavigation-assisted endoscopic procedure to remove the intraventricular hematoma, along with intracranial pressure (ICP) monitoring. After surgery, she recovered smoothly and showed marked neurological improvement.

**Conclusion:**

This case indicates that even brief stays at moderately high altitude may impair cerebrovascular self-regulation and increase stress on blood vessel walls. The resulting instability in blood flow during re-adaptation to lower elevation, combined with a sudden rise in intracranial pressure from a Valsalva-type action such as coughing, could lead to the rupture of susceptible vessels. Medical practitioners should consider this possible cause in cases of neurological emergency where there is a recent history of high-altitude exposure.

## 1 Introduction

Exposure to high-altitude conditions, marked by low oxygen availability, leads to a range of physiological stress responses in the body (Pena et al., 2022). These reactions can include activation of the sympathetic nervous system, raised blood pressure, greater blood flow to the brain, concentration of blood cells, and impaired function of blood vessel linings (Li et al., 2024). Such changes are known to contribute to intracranial bleeding at high altitudes—a problem more frequently seen in people living there long-term or those with conditions like excessive red blood cell production due to altitude (Zhu et al., 2013).

Nevertheless, cerebrovascular incidents affecting short-term visitors to high-altitude areas—particularly those that manifest after returning to lower elevations—have not gained adequate attention in clinical practice (Lagowski et al., 2025). The process of re-adapting to normoxic conditions, often referred to as “de-acclimatization,” involves a reversal of physiological changes acquired at altitude. Rapid return to lower elevation, whether by air or high-speed rail, forces this adaptation to occur within hours rather than days. This abrupt transitional period poses significant risks, including acute instability in cerebral blood flow and autoregulation (Luks and Hackett, 2022).

Coughing, as a common form of the Valsalva maneuver, can abruptly raise pressure within the chest, abdomen, and skull. This sudden rise may cause temporary spikes in blood pressure and reduce blood flow back from the brain. Although it is an uncommon cause, coughing is a recognized trigger of spontaneous bleeding inside the skull (Sallinen et al., 2020).

This article reports a case of primary intraventricular hemorrhage that occurred in a previously healthy young woman following rapid return from moderate high altitude. The hemorrhage was immediately preceded by vigorous coughing, suggesting this Valsalva maneuver served as the final trigger in a cerebrovascular system likely stressed by altitude exposure and rapid environmental transition. We aim to discuss the potential synergistic effects of these factors and enhance clinicians' awareness of this condition.

## 2 Case presentation

### 2.1 Patient information

A 30-year-old, right-handed Han Chinese woman was admitted to the emergency department due to “sudden onset of severe headache and vomiting for 3 h”. She had no significant past medical history, denied history of hypertension, diabetes, coagulopathies, and had no habits of smoking, alcohol abuse, or illicit drug use. There was no family history of early-onset cardiovascular or cerebrovascular diseases.

### 2.2 History and precipitating factors

The patient was a long-term resident of the low-altitude plains in Jiangsu Province. This four-day trip to Xining City at 2,260 m represented her first documented exposure to a high-altitude environment. During her stay, she developed severe and persistent insomnia with fewer than 3 h of sleep per night. She explicitly denied experiencing headache, nausea, dizziness, or other typical symptoms of acute mountain sickness. This isolated sleep disturbance represented her primary manifestation of high-altitude physiological stress.

On the day she got sick, she flew back in the morning to Xi'an, which has a much lower altitude of around 400 m. About 6 h after landing, she experienced a sudden intense coughing fit. The patient denied any preceding respiratory symptoms, allergies, or history of chronic cough. No specific cause for the cough was identified in the emergency evaluation. Right afterward, she was struck by a severe “thunderclap” headache—so extreme she could hardly bear it—along with dizziness, nausea, and several bouts of vomiting yellow stomach fluid. She did not experience seizures, pass out, lose bladder control, or have any weakness in her limbs.

### 2.3 Physical examination on admission

Vital Signs: Temperature 36.5°C, heart rate 62 beats/min, breathing rate 20 breaths/min, blood pressure 136/91 mmHg (the patient reported her usual blood pressure is around 110/70 mmHg). Heart, lung, and abdominal exams showed no notable abnormalities.

Neurological Exam: The patient was conscious but drowsy, with clear speech. Her Glasgow Coma Scale (GCS) score was 14 (E3V5M6). Both pupils were equal, round, and 3.0 mm in diameter, reacting normally to light. Eye movements were full with no nystagmus. Facial sensation was symmetric; forehead wrinkles and nasolabial folds were even; the tongue was midline. Limb muscle size and tone were normal. Muscle strength was rated grade IV in all four limbs (using the Medical Research Council scale). Light touch and pinprick sensation were generally intact. Deep tendon reflexes (biceps, brachioradialis, and knee jerks) were ++ on both sides. Babinski signs were negative bilaterally. Significant neck stiffness was noted (about three finger-widths between chin and chest). Kernig's sign was positive.

### 2.4 Diagnostic investigations

**Figure 1:**
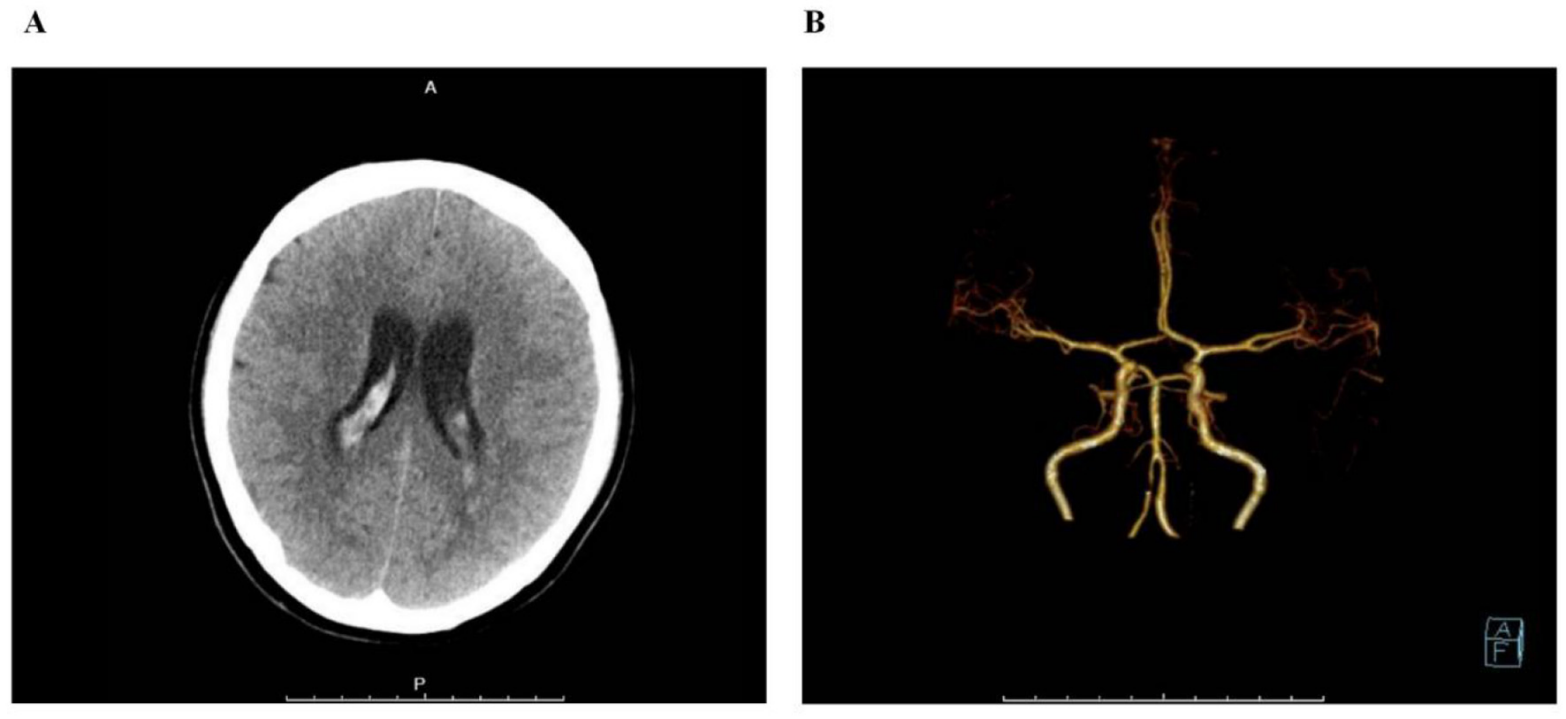
**(A)** Non-contrast CT scan on admission showing hyperdense blood filling the ventricular system. **(B)** CTA 3D reconstruction showing no visible vascular abnormalities.

Non-contrast cranial CT (emergency): Revealed hyperdense cast-like shadows within the lateral, third, and fourth ventricles, suggestive of intraventricular hemorrhage (Figure 1A). No definite intraparenchymal hematoma was seen. Sulci were slightly effaced, indicating possible cerebral swelling.

CT angiography (CTA): The circle of Willis was clearly displayed. Major arteries and their main branches coursed naturally, with smooth walls, no stenosis, dilation, or abnormal protrusions. No definite aneurysms or vascular malformations were identified (Figure 1B).

Laboratory tests: Complete blood count: White blood cells 8.7 × 10^9^/L, Hemoglobin 132 g/L, Hematocrit 39.0%, Platelets 215 × 10^9^/L. Coagulation profile: Prothrombin time 12.5 s, INR 1.05, Activated partial thromboplastin time 32.1 s. Liver and renal function tests, electrolytes, and blood glucose were within normal ranges.

### 2.5 Treatment and outcome

The initial diagnosis was “Primary Intraventricular Hemorrhage with Unknown Cause.” Right after admission, she received complete bed rest, heart monitoring, medication to control bleeding (tranexamic acid), drugs to lower brain pressure (mannitol), neuroprotective treatment, and general support.

**Figure 2:**
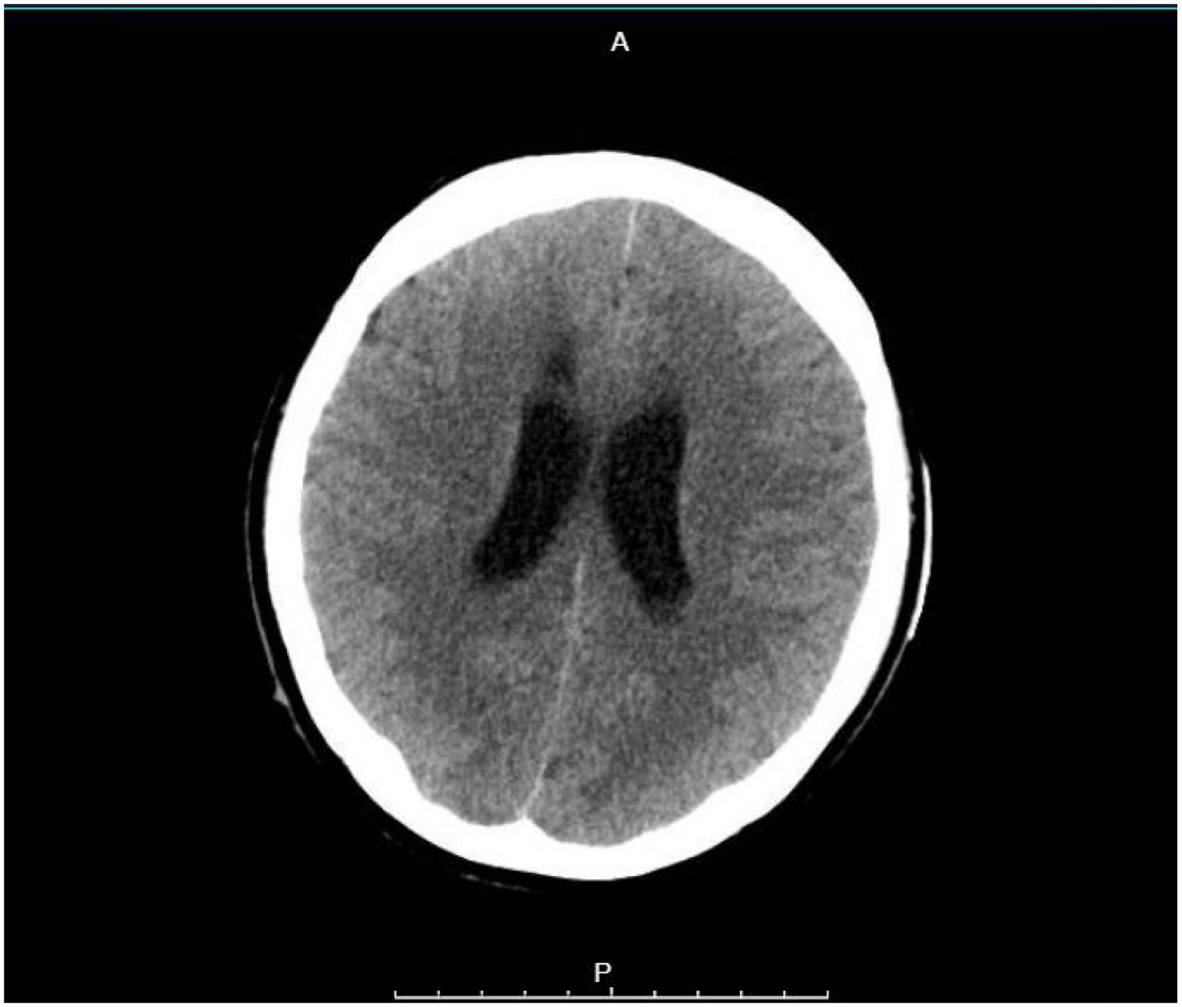
Follow-up non-contrast axial CT scan obtained on the sixth postoperative day after endoscopic evacuation, demonstrating significant clearance of the intraventricular hematoma with restored ventricular anatomy and cerebrospinal fluid pathways.

Because there was a large amount of blood in the ventricles and a real risk of blocked fluid flow and further decline, she had surgery on the second day. Under general anesthesia, doctors used computer-guided endoscopy to remove the clot, placed a drain, and inserted a pressure monitor inside the ventricle. During surgery, the initial intracranial pressure measured upon ventricular access was 28 mmHg, confirming significant intracranial hypertension. Following clot evacuation, the pressure normalized to 10–15 mmHg. During the procedure, many fresh blood clots were found and taken out, which reopened the normal fluid pathways. After surgery, her brain pressure stayed stable, ranging between 10 and 15 mmHg. A repeat CT scan showed most of the bleeding had cleared (Figure 2).

Her headache improved significantly, neck stiffness went away, and within a week, strength returned to normal in all four limbs. She was discharged with a Glasgow Outcome Scale score of 5, meaning she recovered well. She was advised to avoid high-altitude travel in the future and to try to prevent respiratory infections.

## 3 Discussion

**Figure 3:**
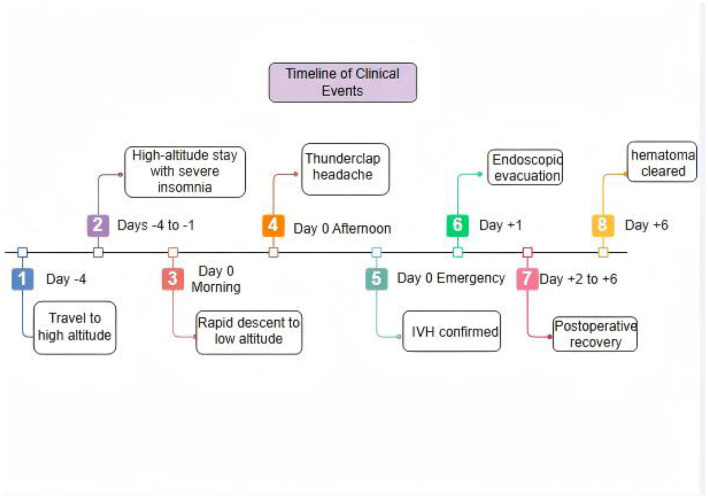
Timeline of clinical events. The timeline illustrates the sequence from high-altitude exposure (Day −4 to −1) with severe insomnia, rapid descent to low altitude (Day 0 morning), the onset of vigorous coughing followed by thunderclap headache (Day 0 afternoon), emergency admission and diagnosis, endoscopic surgery (Day +1), postoperative recovery with follow-up CT on Day +6.

This patient was a young woman with none of the usual risk factors for stroke or bleeding in the brain. The CT angiogram came back normal, effectively ruling out common causes like aneurysms or blood vessel malformations. What stands out is the clear timing between her bleeding event and two specific triggers: a recent short-term stay at high altitude and a forceful coughing episode soon after returning to low elevation (Figure 3). This close sequence of events points to a possible unique mechanism linking altitude changes and coughing to the brain bleed.

### 3.1 High-altitude stress and cerebrovascular vulnerability

The altitude in Xining is about 2,260 m, which is considered moderate. While this isn't high enough to cause serious altitude sickness, the thinner air can still stimulate the body's stress response systems. This may lead to a faster heart rate and higher blood pressure—as seen in this patient, whose blood pressure was 136/91 mmHg at admission compared to her usual 110/70 mmHg (Duo et al., 2023; Parati et al., 2014).

At the same time, the body responds to lower oxygen by widening blood vessels in the brain to improve blood flow. It's also important to note that the patient's significant trouble sleeping—a common issue at altitude—can further increase nervous system activity and affect blood vessel function (Bigalke et al., 2022). Together, these factors likely put extra strain on the blood vessels in her brain over just a few days, making them more vulnerable to injury.

It is noteworthy that, based on our hospital's experience as a primary medical center for high-altitude returnees, similar events have occurred in both males and females. This observation suggests that the described hemodynamic mechanism, which is driven by altitude-related stress and rapid de-acclimatization, represents the principal pathogenic pathway. This pathway operates independently of gender-specific hormonal influences.

### 3.2 Risks during de-acclimatization

Our hospital's location in a major transit hub for travelers returning from high-altitude regions provides relevant epidemiological context. We regularly encounter cases of cerebrovascular events, including intracranial hemorrhage, that occur within 24–48 h following rapid return from high altitude. This pattern is observed not only in air travelers but also in those returning by high-speed rail, suggesting that the critical factor may be the speed of environmental transition rather than transport-specific mechanisms.

Returning to lower altitude by air allows the body to readjust from oxygen-poor to oxygen-rich conditions in just a few hours. This rapid shift requires the blood vessels in the brain to narrow quickly in response to the higher oxygen levels in the blood. Such a sudden change can disrupt the brain's natural ability to regulate blood flow, leaving the vascular system temporarily unstable, and more sensitive. During this period, the brain may struggle to maintain steady circulation, becoming more susceptible to sudden changes in blood pressure.

### 3.3 Cough as the “final trigger”

Coughing can generate a forceful, involuntary Valsalva-like strain distinct from the gentle maneuver recommended for ear pressure equalization. This maximal strain produces an acute and severe surge in intrathoracic and intracranial pressure. When acting upon a cerebrovascular system already compromised by high-altitude stress and rapid de-acclimatization, such a pressure surge may serve as the final trigger for vascular rupture (Canac et al., 2020). For a cerebrovascular system already rendered precarious by high-altitude stress and subsequent de-acclimatization, this sudden pressure overload could be sufficient to rupture a vulnerable vessel wall, most likely a small artery or vein in the subependymal region or choroid plexus. The mechanism shares similarities with that underlying “cough syncope,” albeit with a more severe outcome in this instance.

For the general population, standard ear pressure equalization techniques remain safe. However, individuals exhibiting significant physiological symptoms following rapid return from high altitude, particularly severe insomnia, should be advised to avoid vigorous straining maneuvers during the initial 48 h after descent. This includes suppressing forceful coughing when possible and avoiding heavy lifting or intense breath-holding activities.

### 3.4 Differential diagnosis and clinical implications

This case is compelling because the CTA scan ruled out typical sources of spontaneous brain bleeding, such as aneurysms, arteriovenous malformations, and moyamoya disease. We acknowledge that MRA remains a more sensitive tool for detecting certain vascular abnormalities, and its use could further strengthen the diagnostic certainty (Denby et al., 2020). While it's impossible to completely eliminate the possibility of a very hidden vascular issue, the strong combination of clear triggers and a well- documented timeline makes an environmentally induced cause the most reasonable explanation.

The diagnostic evaluation appropriately focused on identifying structural and major vascular anomalies as immediate causes. Specialized assessments for capillary fragility, such as the Rumpel-Leede test, or formal testing of primary hemostasis through bleeding time measurement were not part of the acute workup. This approach aligns with contemporary standards of care for spontaneous intracranial hemorrhage, where such tests are not routinely indicated. The patient presented with entirely normal basic coagulation parameters and platelet counts, and she exhibited no clinical features suggestive of a systemic bleeding disorder, such as spontaneous bruising or mucosal bleeding. Consequently, a generalized microvascular defect or primary hemostatic dysfunction was considered improbable. The most plausible etiology remains a localized hemodynamic insult to a vulnerable cerebral vessel, occurring within the specific physiological context of high-altitude stress and rapid de-acclimatization. The quantitative intracranial pressure measurement of 28 mmHg during surgery objectively documented the substantial intracranial hypertension in this case. This finding, along with the rapid normalization of pressure after endoscopic evacuation, provides important physiological data for recognizing and managing similar presentations.

There are three key clinical implications from this case: first, individuals experiencing brain blood vessel issues at high altitude are not limited to long-term residents—short-term visitors may also be susceptible; second, the risk persists beyond the high-altitude exposure itself and extends into the “re-adaptation” period after returning to lower elevations; and third, obtaining a detailed travel history should become a routine component of the clinical assessment for any patient presenting with unexplained intracranial hemorrhage.

## 4 Conclusion

This case, supported by our institutional experience, suggests that rapid return from high altitude—regardless of the specific mode of transport—may create a critical window of cerebrovascular vulnerability. The risk appears heightened in individuals who exhibit signs of physiological stress during altitude exposure, such as severe insomnia. In such situations, common actions like coughing could serve as a final trigger for hemorrhage. While this single case cannot prove a definitive cause-and-effect relationship—and larger case-control studies would be needed to confirm the association—the findings still offer practical insight for neurologists, emergency doctors, and travel medicine specialists. Recognizing this possible link may help identify at-risk individuals earlier, particularly those with recent high-altitude exposure who develop symptoms like severe insomnia, and improve guidance regarding the timing and manner of return travel.

## Data Availability

The original contributions presented in the study are included in the article/supplementary material, further inquiries can be directed to the corresponding authors.
